# Application of Sephadex LH-20 for Microdetermination of Dopamine by Solid Phase Spectrophotometry

**DOI:** 10.5402/2012/216068

**Published:** 2012-11-28

**Authors:** Mehdi Taghdiri, Arash Mohamadipour-taziyan

**Affiliations:** Department of Chemistry, Payame Noor University, P.O. Box 19395-3697, Tehran, Iran

## Abstract

A sensitive spectrophotometric method for the determination of dopamine was carried out without any separation steps. Bromocresol green is adsorbed on Sephadex LH-20 gel but the sorption decreases in the presence of dopamine due to ion-pair formation between bromocresol green and dopamine in solution. This attenuation was used to the microdetermination of dopamine by measurement of absorbance of the solid phase (Sephadex LH-20 gel) in a 1.0 mm cell at 625 nm. Dopamine could be determined in the concentration range of 0.4–1.6 **μ**g mL^−1^ (10-mL Sample volume) with a relative standard deviation (RSD) of 0.03% (*n* = 4). The detection limit was obtained, 0.26 **μ**g mL^−1^ (1.7 **μ**M). The method was used for determination of dopamine in pharmaceutical injection sample and satisfactory result was obtained.

## 1. Introduction

Dopamine (DP) has been used for treating all kinds of shock syndromes. It is very important to find a simple and sensitive method to determine the content of DP in clinical medicine. Pharmaceutical quality standard of many countries describes a nonaqueous titration method for determination of DP in injection, for example, the 1990 edition of Chinese pharmacopoeia and USP XXI. HPLC method was described for the determination of DP in USP 24 and BP 2000 with expensive apparatus [1]. Chemiluminescence has also been applied to determine DP; however, chemiluminescence reactants have hardly found extensive applications due to the instability of the oxidation reagents [2, 3]. Electrochemical detection methods have also been introduced since these are the best ways to directly determine DP without any separation steps of samples. The existence of ascorbic acid (AA) is the main obstacle in electrochemical detection system since AA is oxidized at similar potentials to DP at conventional electrodes. Therefore, modified electrodes have been used to simultaneously detect both DP and AA at different potentials [4–12]. The spectrophotometry has also been used for determination of DP with the use of organic compounds that react with DP [13–20]. However, in this work, the determination of DP is carried out with inhibition of the sorption of bromocresol green (BCG) on a solid phase from an aqueous solution, and the subsequent measurement of absorption, directly in the solid phase. Solid phase spectrophotometry (SPS) in visible region has been less used for determination of organic compounds [21]. We present an innovative work to use SPS in visible region for determination of DP. A higher molar absorptivity was obtained by this proposed method than other spectrophotometric methods.

**Figure 1:**
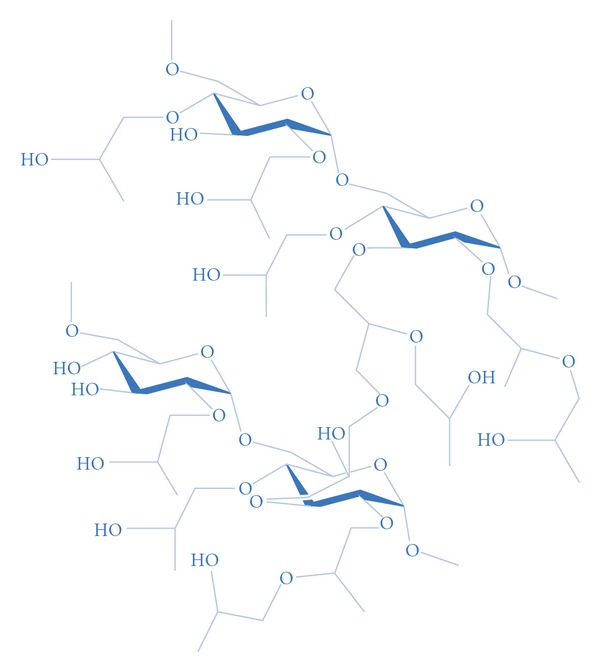
Partial structure of Sephadex LH-20.

Dopamine forms an ion-pair with BCG in solution and therefore, the sorption of BCG on the solid phase decreases. We used the Sephadex LH-20 as solid phase. Sephadex LH-20 medium is based on hydroxypropylated dextran that has been cross-linked to yield a polysaccharide network (Figure 1).

## 2. Materials and Methods

### 2.1. Materials and Solutions

Citric acid, sodium hydroxide, potassium hydroxide, sodium acetate, sodium ascorbate, acetic acid, and bromocresol green (all from Merck) were of the highest purity available and were used as received. 3-Hydroxytyramine hydrochloride (Dopamine, 99%) was purchased from Acros Organics.

The Sephadex LH-20 gel (mesh 25–100 mm) (Aldrich) in its original dry state without pretreatment was used as solid support. Dowex 1-X8 (200–400 mesh) anion exchange resin (Bio-Rad) was used in the chloride form for removal of ascorbate interference.

The stock solution was prepared by dissolving an appropriate amount of DP in water to obtain a concentration of 3.0 × 10^−4^ M. Citrate buffer, pH 4.0 was prepared by titrating aqueous 0.1 M disodium citrate (19.2 g citric acid + 200 mL of 1 M NaOH/1000 mL H_2_O) with 0.1 M HCl. Bromocresol green was dissolved in 4.0 × 10^−4^ M NaOH and was diluted to 100.0 mL with the pH 4.0 citrate buffer to produce a 1.0 × 10^−4^ M solution. Buffer solutions and bromocresol green stock solutions were adjusted to an ionic strength of 0.2 with KCl. The pharmaceutical injection sample (Dopadic) was from Caspian Tamin Pharmaceutical Co., Iran. 

### 2.2. Apparatus

Weighing of materials was performed by using an analytical balance model Sartorius MCBA 100 with precision of ±0.0001 g. pH measurements were carried out with a Metrohm 691 pH-meter. A GBC spectrophotometer model Cintra 6 was used for spectrophotometric measurements.

### 2.3. Procedures

A 10 mL sample containing 0.4–1.6 *μ*g mL^−1^ of DP was transferred to a 100 mL Beaker and then 4 mL of 1.0 × 10^−4^ M BCG was added. The mixture was stirred mechanically for 4 min. Then 70 mg of Sephadex LH-20 (25–100 mesh) gel was added and the mixture was stirred mechanically for 5 min and the coloured gel was collected by centrifuge and, using a little pipette, packed into a 1 mm cell together with a small volume of the filtrate. The cell was centrifuged at 2500 rpm for 1 min. A blank solution containing all the reagents except DP was prepared and treated in the same way as the sample. The absorbance (really attenuation) of Sephadex LH-20 gel was measured at 625 nm (corresponding to the absorption maximum of the BCG) and 800 nm (the latter is in the 700–850 nm range, where only the gel “absorbs” light) and compared with a 1 mm cell packed with gel equilibrated with blank solution. The absorbance difference between sample and blank provides an estimation of the net absorbance.

DP injection solution (200 mg per 5 mL) was appropriately diluted with water to get the required concentration of the drug, and then the general procedure was followed. The amount of DP was calculated from a calibration graph.

## 3. Results and Discussion 

### 3.1. Absorption Spectra

**Figure 2:**
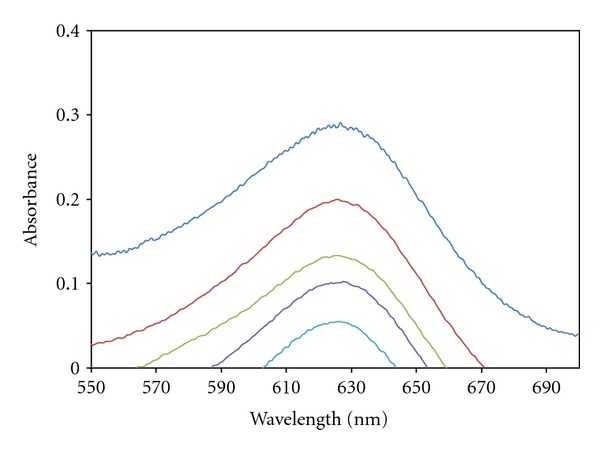
The decreases of absorbance of BCG in the Sephadex LH-20 phase in the presence of the various amounts of DP (1-mm cell, similarly packed with Sephadex LH-20 equilibrated with water as reference). [BCG] = 10^−4^ M, 70 mg Sephadex LH-20; from up to down [DP] = 0.0, 0.4, 0.8, 1.2 and 1.6 *μ*g mL^−1^.

The BCG color reagent (pK_a_ = 4.66) occurs in two acid-base forms in weakly acidic aqueous solutions with the absorption maximum at 430 nm (BCGH^−^ form) and 615 nm (BCG^2−^) [22]. When this triarylmethane dye forms ion-pair with DPH^+^ in citrate buffer solution at pH 4.0, it is not adsorbed on Sephadex LH-20 gel. Therefore, the absorbance difference between solid phase prepared in the absence and in the presence of DP is proportional to DP concentration in solution (Figure 2).

### 3.2. Optimization of Conditions

#### 3.2.1. pH Dependence

**Figure 3:**
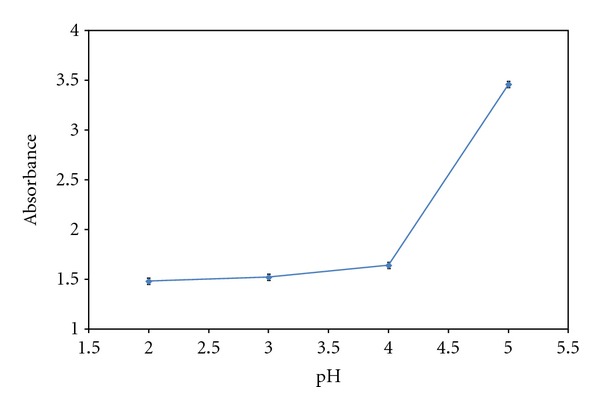
Influence of pH on the absorbance of BCG in solid phase. Conditions: 1.2 *μ*g mL^−1^ of DP; [BCG] = 10^−4^ M; 70 mg Sephadex LH-20; sample volume 10 mL (air as reference).

Optimum pH for the formation of ion-pair and fixation of BCG on Sephadex LH-20 falls below 4.0 (Figure 3). At pH values of above 4.0 the net absorbance of the solid phase increased due to a decrease in the formation of ion-pair. In pH 3.0, the monoanionic form of BCG is predominant and then solid phase is yellow with the absorption maximum at 435 nm but the solid phase is green in pH 4.0 with the absorption maximum at 625 nm. We chose pH 4.0 as the optimum pH value for the procedure because the absorbance of solid phase was further from UV region. The best of the buffer systems examined was citric acid-citrate (pH = 4).

#### 3.2.2. Other Experimental Conditions

**Figure 4:**
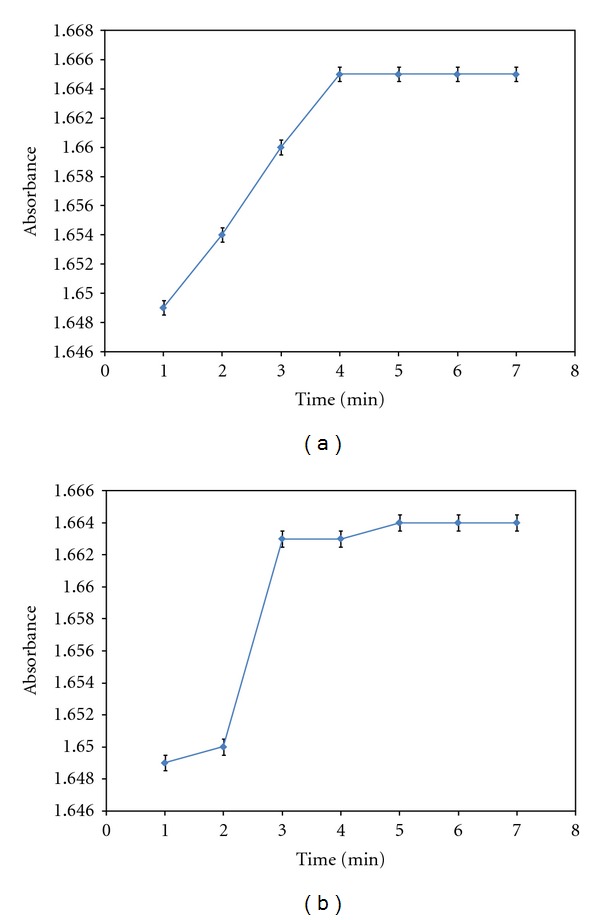
Influence of stirring time on the absorbance of BCG in solid phase. Conditions: 1.2 *μ*g mL^−1^ of DP; [BCG] = 10^−4^ M; pH = 4.0; 70 mg Sephadex LH-20; sample volume 10 mL (air as reference). (a) before adding of Sephadex LH-20 and (b) after adding of Sephadex LH-20.

The optimum stirring time before and after adding of Sephadex LH-20 were 4 min and 5 min, respectively, (Figure 4). The fixed BCG is stable for at least 80 min after equilibration. Repeatability of the method is improved if the cells packed with the solid phase are centrifuged before spectrophotometric measurements are taken. The centrifugation time used here was 1 min at 2500 rpm. Sephadex LH-20 of between 25 and 100 mg allow adequate working conditions. A decrease in the amount can result in operational difficulties. For all measurements 70 mg of Sephadex LH-20 was used as a compromise between maximum sensitivity and ease of operation.

### 3.3. Analytical Data

The calibration graph is reasonably linear for the concentration ranges 0.4–1.6 *μ*g mL^−1^ of DP for the 10 mL sample system. The analytical parameters are shown in Table 1.

Repeatability was measured for a series of four independent determinations containing 1.2 *μ*g mL^−1^ of DP and was 0.03%.

The sensitivity, expressed as molar absorptivity, of the proposed method is compared in Table 2 with those of published spectrophotometric methods. The higher sensitivity of the proposed method is apparent.

### 3.4. Interference

An antioxidant, sodium metabisulphate, and sodium chloride that are commonly present in the DP injection, and also commonly used excipients such as starch, talc, glucose, lactose, dextrose, and magnesium stearate, did not interfere.

The most serious interference in the determination of DP in biological samples is ascorbate anion [4–10, 23, 24]. However, this interference can be removed by treating the sample using Dowex 1-X8 anion exchange resin before determining of DP by the SPS method. DP is not adsorbed on Dowex 1-X8 because it is as cationic form (Table 3).

### 3.5. Determination of DP in Pharmaceutical Sample

The method has been applied to the determination of DP in pharmaceutical sample by calibration curve method (ΔA versus concentration of DP). The result obtained, summarized in Table 4, shows a good agreement with the composition value indicated by the supplier. It should be noted that our purpose of this determination is not to assay this pharmaceutical sample but is to indicate the ability of the SPS method in microdetermination of DP. The detection limit of the SPS method is 0.26 *μ*g mL^−1^ (1.7 *μ*M).

## 4. Conclusions

Dopamine at the microgram level can be determined efficiently with BCG using the Solid-Phase Spectrophotometry technique without expensive apparatus. The proposed method has been applied to the determination of DP in pharmaceutical sample with good result. The method is simple and more sensitive as compared to others commonly used at the microgram level. The method does not use organic solvents, extra organic compounds as reactants and pretreatment of the sample. Therefore, the method is environmentally friendly and can be considered as a green analytical method. It has been demonstrated that the interference of ascorbate, if it exists in the sample, can be removed by anion exchanger resin and hence, the method can be appropriate for the determination of DP in biological samples.

## Figures and Tables

**Table 1:** Analytical parameters.

Intercept	0.0897
Slope (mL *μ*g^−1^)	0.1164
Linear dynamic range (*μ*g mL^−1^)	0.4–1.6
Correlation coefficient	0.9904
RSD (%)	0.03
Detection limit (*μ*g mL^−1^)	0.26
ΔA for sample	0.1096 ± 0.0012^a^

^a^Average of three determinations.

**Table 2:** Comparison of sensitivity of some spectrophotometric methods for the determination of dopamine.

Method	Molar absorptivity (L·mol^−1^·cm^−1^)	Reference
Reaction with 1,2-naphthoquinone-4-sulfonate	2.78 × 10^3^	[14]
Diazotised with sulphamic acid	4.2 × 10^3 ^	[16]
Isoniazid in the presence of *N*-bromosuccinimide	6.47 × 10^3 ^	[15]
Thiosemicarbazide	2.4 × 10^4 ^	[20]
Potassium ferricyanide-Fe(III)	3.2 × 10^4 ^	[19]
Diazotised sulphanilamide in the presence of molybdate	5.39 × 10^4^	[17]
Proposed method (solid phase spectrophotometry)	1.78 × 10^5^	This work

**Table 3:** Effect of ascorbate on the determination of 1.2 *μ*g mL^−1^ of dopamine.

Ascorbate content (*μ*g mL^−1^)	Absorbance of solid phase^a^
0	1.664
0.4	1.644
0.4	1.664^b^
0.6	1.664^b^

^a^Air as reference, ^b^After removal of ascorbate by Dowex1-X8.

**Table 4:** Determination of dopamine in pharmaceutical sample.

Drug	Nominal content of DP after dilution (*μ*g mL^−1^)	Obtained content of DP by SPS (*μ*g mL^−1^)
Injection (Marketed by Caspian Tamin Pharmaceutical Co.)	1.2	1.11 ± 0.01^a^

^a^Average of three determinations.
